# Perspective: Contribution of Epstein–Barr virus (EBV) Reactivation to the Carcinogenicity of Nasopharyngeal Cancer Cells

**DOI:** 10.3390/cancers10040120

**Published:** 2018-04-17

**Authors:** Chung-Chun Wu, Chih-Yeu Fang, Sheng-Yen Huang, Shih-Hsin Chiu, Chia-Huei Lee, Jen-Yang Chen

**Affiliations:** 1 National Institute of Cancer Research, National Health Research Institutes, Zhunan 350, Taiwan; chungcwu@nhri.org.tw (C.-C.W.); 030735@nhri.org.tw (S.-Y.H.); d96445003@ntu.edu.tw (S.-H.C.); chlee124@nhri.org.tw (C.-H.L.); 2 Department of Pathology, Wan Fang Hospital, Taipei Medical University, Taipei 116, Taiwan; phildts@gmail.com; 3 Department of Microbiology, College of Medicine, National Taiwan University, Taipei 100, Taiwan

**Keywords:** Epstein–Barr virus, reactivation, lytic cycle, genomic instability, nasopharyngeal carcinoma

## Abstract

Nasopharyngeal carcinoma (NPC) is a squamous cell carcinoma derived from the epithelium of the post-nasal cavity, with a unique geographic and ethnic distribution. Epstein–Barr virus (EBV) is an etiological agent of NPC, but how it contributes to carcinogenesis is not completely clear. Although it is thought that EBV latency participates in the development of NPC, increasing evidence reveals that the lytic cycle also plays an important role in the carcinogenic process. In this review, we summarize our recent studies on how EBV reactivation causes genomic instability and accelerates tumorigenesis in epithelial cells. The roles of three lytic genes, namely, *BRLF1*, *BGLF5* and *BALF3*, in this process are also introduced. Moreover, blocking EBV reactivation using natural compounds may help delay the progression of NPC tumorigenesis. These studies provide a new insight into NPC carcinogenesis and raise the possibility that inhibition of EBV reactivation may be a novel approach to prevent the relapse of NPC.

## 1. Introduction

Epstein–Barr virus (EBV) infection, consumption of nitroso-compounds, and genetic factors have been implicated in the carcinogenesis of nasopharyngeal carcinoma (NPC) [1,2]. Individuals with high levels of antibodies against EBV have been shown to have a greater risk of NPC onset [3,4,5]. These findings suggest that EBV may contribute to the carcinogenesis of NPC, including initiation and relapse. 

Although years of study led to the proposal that latent EBV infection contributes to the carcinogenesis of NPC [6], on the basis of epidemiological studies, most adults in Taiwan are EBV carriers but only a relatively small number develop NPC. These aspects prompted us to study whether EBV reactivation plays a more important role in the carcinogenesis of NPC.

Extensive studies have been carried out on the contributions of EBV latent genes in the carcinogenesis of NPC (reviewed in [6,7,8]). On the other hand, although lytic genes have been associated with EBV carcinogenetic effects, such as BZLF1 in lymphoblastoid cell lines [9], BCRF1 in human B lymphocytes [10], and BARF1 in gastric cancer ([11] and reviewed in [12]), fewer investigations have been conducted on the contribution of EBV lytic genes to the tumorigenesis of NPC. 

There is no good cell culture model available to study the effects of EBV on the initiation of carcinogenesis in normal nasopharyngeal cells. In NPC patients, before relapse, antibodies against EBV elevate again [13], possibly raised by antigens expressed after EBV reactivation in residual NPC cells containing latent EBV. We elected to study the effects of EBV on the carcinogenesis of NPC cells. Our findings may suggest a model whereby residual NPC contribute to the relapse of NPC after remission following therapy. Relapse is the major cause of mortality of NPC.

## 2. Reactivation of EBV Has a Significant Carcinogenic Effect on the Genomic Instability (GI) and Tumorigenesis of NPC Cells

GI is one of the hallmarks of cancer [14] and is considered to contribute to cancer development. Using micronucleus (MN) formation as a marker of GI [15], we first demonstrated that EBV reactivation, induced by 12-*O*-tetradecanoylphorbol-13-acetate (TPA) and sodium n-butyrate (SB), caused GI and had tumorigenic effects on NA cells, an EBV-positive cell line [16] derived from EBV-negative NPC-TW01 cells [17,18]. A single treatment induced DNA double-strand breaks and formation of MN in NA cells. Recurrent treatment resulted in an increase in chromosome aberration and in the invasiveness and tumorigenicity of NA cells. These results indicate that recurrent EBV reactivation may contribute to the accumulation of GI and promote tumorigenic progression of NPC cells.

## 3. *BGLF5* is the Strongest Inducer of Micronuclei Formation and DNA Damage

EBV is a herpesvirus with the typical replication cycle of latency and lytic infection. Infection of B cells with EBV results in latency; upon induction with TPA, the virus enters the lytic cycle, and immediate early genes, early genes, and late genes are expressed sequentially, with the formation of viral particles and the lysis of the host cells [19]. To determine which genes may be involved in the induction of GI, we expressed several EBV lytic genes, including *BZLF1*, *BXLF1*, *BALF2*, *BKLF3*, and *BGLF5* in TW01 cells. The formation of MN and phosphorylation of H2X were examined, and *BGLF5* was found to have the greatest effect on their induction [18]. Further study indicated that *BGLF5* alone is able to induce DNA damage and repress the transcription of DNA repair enzymes [20].

## 4. BALF3 Mediates Genome Instability and Progressive Malignancy in NPC Cells

Despite *BGLF5* expression inducing the strongest GI for NPC TW01 cells, we had difficulty demonstrating its ability to enhance cell tumorigenicity, because of its cytotoxic effect (unpublished result). We investigated the function of BALF3, a terminase, which has nuclease activity and acts in the production of mature EBV virions during the lytic cycle [21]. Recurrent expression of BALF3 in NPC TW01 cells induced genomic copy number aberrations and tumorigenic features, including cell migration, cell invasion, and spheroid formation. In addition, after recurrent induction of BALF3, the cells developed into large tumor nodules when inoculated into NOD/SCID mice [22].

## 5. BRLF1 Induces Genomic Instability and Progressive Malignancy in NPC Cells

*BGLF5* and *BALF3* are early genes in the EBV lytic cycle, and we wished to determine whether EBV immediate early genes contribute to the induction of GI and the enhancement of tumorigenicity in NPC cells. We first examined the effect of *BZLF1*, an immediate early gene. Its expression in NPC TW01 cells did not lead to a significant increase in MN formation [18]. This suggests that *BZLF1* may not play a role in the induction of GI in NPC cells. We further investigated *BRLF1*. Surprisingly, we found that *BRLF1* induced chromosome mis-segregation and GI in NPC TW01 cells. Further experiments indicated that Erk signaling is important for *BRLF1* to exert its function. Chromosome aberrations and tumorigenic features increased with rounds of *BRLF1* expression, and the cells developed into large tumor nodules in mice [23].

## 6. EBV Reactivation by Chemical Carcinogens May Contribute to the Carcinogenesis of NPC Cells

The consumption of nitroso compounds has been considered to be an important factor contributing to the carcinogenesis of NPC [1]. We were interested in determining whether nitroso compounds can induce GI and contribute to the carcinogenicity of NPC cells. Nitroso compounds are a group of compounds containing a nitroso group bound to a nitrogen atom. Dietary intake of nitroso compounds has been associated with NPC [24]. We chose to study *N*-methyl-*N*′-nitro-*N*-nitrosoguanidine (MNNG, a nitrosamide). EBV reactivation was observed in NA cells after treatment with MNNG, and the reactive oxygen species (ROS) scavenger *N*-acetyl-l-cysteine (NAC) inhibited this reactivation. Therefore, ROS were found to play an important role in the reactivation of latent EBV [25]. In addition, a low dose of MNNG (0.1 μg/mL) had a synergistic effect with TPA/SB in enhancing EBV reactivation [26], and consequent increases in GI and tumorigenicity were observed in NPC cells treated with MNNG, alone or in combination with TPA/SB. The combination exerted a very strong synergistic effect [27].

## 7. Inhibition of EBV Reactivation May Help Prevent the Malignant Progression of NPC Cells

Because EBV reactivation plays an important role in the carcinogenesis of NPC cells, we sought agents which could block EBV reactivation. Sulforaphane (SFN), a histone deacetylase (HDAC) inhibitor, was found to inhibit EBV reactivation in NA cells treated with TPA/SB. A reporter assay indicated that SFN inhibited the immediate-early gene *BRLF1* but not *BZLF1* [28]. Luteolin (3,4,5,7-tetrahydroxyflavone), a natural flavonoid, blocked EBV reactivation in NA cells treated with TPA/SB by repressing SpI binding to the promoters of the immediate early genes *BZLF1* and *BRLF1* [29]. Apigenin, another flavonoid, was shown to inhibit the reactivation of EBV in NA cells by blocking the *BZLF1* and *BRLF1* promoters [30]. In a mouse study, tumorigenicity induced by EBV reactivation in NPC cells was profoundly decreased following luteolin administration [31]. These results suggest that inhibition of EBV reactivation is a novel approach to prevent the relapse of NPC.

## 8. Perspective

**Figure 1:**
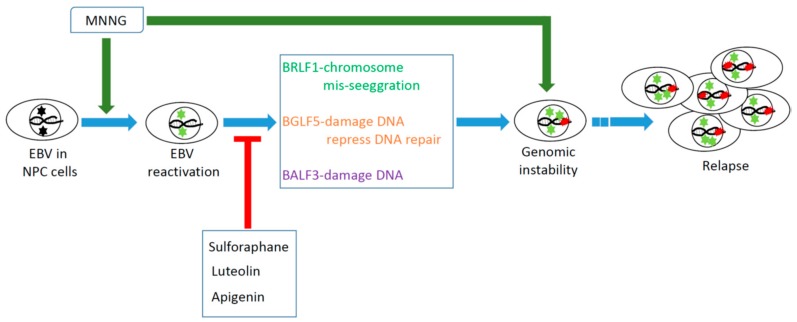
Epstein–Barr virus (EBV) reactivation induces genomic instability and subsequently causes the relapse of nasopharyngeal carcinoma (NPC). *N*-methyl-*N*′-nitro-*N*-nitrosoguanidine (MNNG).

As shown in Figure 1, EBV reactivation contributes to the GI and tumorigenesis of NPC cells. EBV early genes *BGLF5* and *BALF3* and the immediate-early gene *BRLF1* play important roles in the induction of GI and enhancement of tumorigenesis of NPC cells. Agents that inhibit EBV reactivation in NPC cells may be useful for chemoprevention of NPC relapse occurring after treatment.
